# Implementation of a Humanoid Robot as an Innovative Approach to Child Life Interventions in a Children’s Hospital: Lofty Goal or Tangible Reality?

**DOI:** 10.3389/fpsyg.2021.639394

**Published:** 2021-04-19

**Authors:** Tanya N. Beran, Jacqueline Reynolds Pearson, Bonnie Lashewicz

**Affiliations:** ^1^Department of Community Health Sciences, Cumming School of Medicine, University of Calgary, Calgary, AB, Canada; ^2^Alberta Children’s Hospital, Calgary, AB, Canada

**Keywords:** hospital, innovation, child life, pediatric, support (accompaniment), robotics

## Abstract

**Introduction:**

This study reports the findings on how Child life specialists (CLSs) implemented an innovative approach to providing therapeutic support to pediatric patients.

**Methods:**

Part of a larger study that uncovered themes about CLSs’ experiences while working with MEDi^®^, this study reports the reflections that CLSs have about the process of implementation. Seven CLSs participated in semi-structured interviews. Content analysis was conducted on interview data and three themes were generated.

**Results:**

The first was in regards to the adoption process whereby CLS challenges, successes, and surprises were revealed. Second, CLSs explained how using MEDi^®^ aligned with the roles and responsibilities of their profession. The third area of understanding was in CLS explanation of the friendly emotional impact MEDi^®^ seems to have on the hospital environment.

**Conclusion:**

Child life specialists are encouraged to use the MEDi^®^ robot to support children at the bedside.

## Introduction

The need for new or better ideas that add value to hospital care is undeniable (do Carmo Caccia-Bava et al., 2009; Mettler et al., 2017). Indeed, the call for hospital care innovation is widespread and channeled through a variety of venues including conferences, journals, popular magazines, news reports, foundations, and from within hospitals themselves. Defined as the creation of a product or service that results in meaningful enhancements (Cresswell et al., 2018), innovation is the goal of many projects. Most projects do not, however, transform into sustained improvements (MacNeil et al., 2019). In this article, we present a study of how a group of hospital healthcare professionals – namely, child life specialists – implemented and sustained an innovation in the form of incorporating a humanoid robot into their daily practice.

In 2009, the term “innovation implementation failure” was coined to denote slow quality improvement in healthcare (Rangachari, 2018). In fact, “The sluggishness at the health organizational level was particularly striking in light of the increasing availability of evidence-based practices for improving patient outcomes; and the growing momentum toward public reporting of hospital quality” (5, p. 2). Reluctance to implement innovation is even more pervasive in pediatric hospital settings (Maltese and Henrich, 2019). An array of organizational, personal and professional reasons limit the implementation and sustainability of innovation in hospital settings (Nembhard et al., 2019) and key among these reasons is the lack of engagement of frontline workers (Zuber and Weberg, 2020). A frontline worker mindset that is inclined toward active participation and risk taking in learning about, and trying, the innovation is more likely to produce sustained positive change.

One area of healthcare where receptiveness to risk taking and innovation is distinctly significant is within the practice realms of child life specialists. Child life specialists (CLSs) provide individualized hands-on support through education, therapeutic play, and preparation for medical procedures to children and families to reduce distress in healthcare environments. Although they typically work in hospital settings, CLS skills and training are also applicable to community organizations that support the health and well-being of children to help them cope with stress and trauma in settings such as hospices, dental care offices, schools, specialized camps and funeral homes. Descriptions of their daily practice reveal how CLSs are continually challenged to be innovative in meeting ever changing needs of a diverse array of children and their families (Association of Child Life Professionals, 2021). CLSs must collaborate with a variety of healthcare professionals and find ways to apply evidence-based, developmentally appropriate, and psychologically informed strategies to comfort children who are facing any type of medical condition. Such job demands may contribute to a receptiveness on the part of CLSs to try new tools and approaches. Thus, for this study, we collaborate with CLSs using an innovation in the form of a humanoid robot to be incorporated into daily practice.

Originating in factory production, robots are now at work alongside humans in socially complex and high-pressure environments such as hospitals. Robots are used for dispensing medication, delivering materials throughout the hospital, sanitizing surfaces, carrying heavy loads, moving patients, assisting with surgery, and completing minor administrative tasks (Mettler et al., 2017). In addition to providing these forms of physical assistance, there is another group of robots known as socially assistive robots. These types of robots are designed to interact with people and promote social engagement, coaching, and communication (Rabbitt et al., 2015). Despite initial attempts to bring socially assistive robots into healthcare for purposes such as encouraging weight loss and promoting exercise, evidence of effectiveness is limited (Rabbitt et al., 2015). Moreover, these robots have been used primarily with senior patients or children outside of the hospital environment. Robots had not been used at the bedside to support pediatric care until 2011 (Pearson and Beran, 2018). Based on our initial findings that children assign humanistic qualities to robots and even believe robots to be alive (Fior et al., 2010; Beran et al.,2011a,b), our research team at the Alberta Children’s Hospital examined the impact of using a humanoid robot. We selected the NAO robot (SoftbankRobotics), programmed it with cognitive behavior strategies, and named it MEDi^®^ to help children manage vaccinations. We chose this robot because of its endearing appearance (Mubin et al., 2016), versatile capabilities, and affordability. Among other behaviors, MEDi^®^ provides friendly distraction and teaches breathing as a coping strategy to relax (Jibb et al., 2018). When MEDi^®^ demonstrated these behaviors while interacting with children during vaccinations, they experienced lower pain and seemed happier than children who were vaccinated without MEDi^®^ (Beran et al., 2013, 2015). We conducted a similar study comparing pain reports from children having blood tests with and without the friendly distraction of MEDi^®^ and we found similar reports from parents (Manesh et al., 2014). Di Nuovo et al. (2020) also found that some children who interacted with the NAO robot appeared to follow the majority of its instructions before a blood test. Studies with MEDi^®^ used during IV starts, tube removals, dressing change and other procedures all show that children experience lower levels of distress with MEDi^®^ (Farrier et al., 2019; Manaloor et al., 2019).

In this article, we present details of how CLSs at a mid-Western Canadian hospital responded to the research evidence of the impact of the MEDi^®^ robot by incorporating MEDi^®^ as an intervention into their daily practice. We illustrate steps of gradual implementation of MEDi^®^ during visits with families, how CLSs elaborated their use of MEDi^®^ during these interactions, how MEDi^®^ has impacted the hospital environment, and CLSs’ ideas about their future use of MEDi^®^. Our purpose in providing this illustration is to contribute to understandings of the process of implementing and sustaining innovation in a pediatric care hospital setting.

## Materials and Methods

We used an instrumental case study approach to focus on an issue – implementing and sustaining of an innovation in a pediatric care hospital setting. We selected a bounded case – a team of CLS professionals with experience using a particular innovation to illuminate this issue. Lysaker and Buck (2006) point out that a key element of instrumental case study is the provision of detailed contextual information, as context is vital to understanding complex issues of real life situations. As such, below we provide details about the hospital setting at which our study was conducted along with a description of the hospital’s CLS team from which our participants were recruited. At the same time, information about the case *per se* plays a supportive role as the means through which we are able to understand a general issue – in our case, implementing and sustaining of an innovation – beyond the case (Baxter and Jack, 2008).

### Setting and Participants

Participants are CLSs working at the Alberta Children’s Hospital, this province’s most modern children’s hospital, and at the time of opening in 2007, North America’s most state-of-the-art pediatric healthcare facility. This 120 bed free standing tertiary care site represents a family centered approach right from the early days of its design and continuing onto its day to day operations. During the initial planning, children from the community contributed drawings of what they wanted their new hospital to look like, and architects paid attention, noting that children always drew their buildings with large window panes and bright colors. This style was then incorporated into a colorful toy-like design. Not only was consultation from families sought regarding the look of the new hospital but also about practical aspects such as the smell. Families said that they did not want that “hospital smell” and this input was taken so seriously that an innovative ventilation system that turns air over frequently enough to prevent the concerning scent was implemented. The value of play is prominent throughout the indoor and outdoor spaces surrounding the hospital. Developmental and therapeutic activity playrooms are found on each inpatient unit and activities to facilitate socialization with other children are offered regularly by the Child Life and Therapeutic Arts team. Activities include art, music and horticultural therapy programs.

Within this environment, the hospital’s mission is to prioritize research and innovation to achieve excellence in patient care. The MEDi^®^ robot project serves as an example of innovation and an approach to research about improving care. MEDi^®^’s implementation has been inspired by, and models, this strategic initiative. Care at the hospital is also guided by a hospital wide effort to make sure children are comfortable and feel positive about their medical experiences. MEDi^®^ is a tool pediatric healthcare professionals, most frequently CLSs, can adopt to enhance their approach to demonstrating this commitment in the work they do with patients and families, particularly when it comes to pain management.

Alongside the organization’s cultural influences of innovation and commitment to providing the best care possible, is the work of the CLSs team. At the time this research was conducted, the CLS team consisted of four full time equivalent positions designated to each of the inpatient and ambulatory areas, one to the mental health program, and one to the rehabilitation and education program. CLS hours are also allocated to the palliative care and hospice program, and the emergency department. CLSs work with all ages of pediatric patients from newborns to adolescents up to age 18 years striving to meet the developmental needs of each individual child and their families. Siblings of patients are also offered specialized support in the palliative care program. Paid fellowships, student internships, and supervised volunteers also support all areas of the Child Life program.

### Participant Team Context and Experience With MEDi^®^

In 2014, the CLS team attended a research presentation, led by the first author, on how the MEDi^®^ robot reduced children’s pain during flu vaccination and blood tests. Implications of these results for other areas of the hospital were then discussed, the CLSs expressed interest in trying the robot, and a decision was made by the hospital’s management team to purchase four MEDi^®^ robots. Despite having no previous experience with how MEDi^®^ could be implemented into daily child life practice, CLSs were interested in learning about, and experimenting with, using MEDi^®^. They were shown the various behaviors the robot could play and discussions followed about how these behaviors could be applied for therapeutic support. For example, MEDi^®^ dances to popular music, which can be used to distract children and help them relax. MEDi^®^ also greets each child by his or her name to build rapport, and talks about having experienced the same medical procedure that the children will experience as a means of normalizing the situation. For a full description of MEDi^®^’s behaviors, see Pearson and Beran (2018). The specifications of the robot can be found on the manufacturer’s website^1^. Based on the research results and the CLSs’ interest, the CLS team leader encouraged the CLSs to commence incorporating MEDi^®^ in various areas throughout the hospital. Specific plans for the location and purpose of using MEDi^®^ had yet to be determined. CLSs who were interested in trying MEDi^®^ and who saw a possible fit for their patients took the initiative to try the robot. Four of the thirteen CLSs attempted to use MEDi during this early phase of implementation on the units where they normally worked (e.g., medical day treatment and inpatient units). CLSs observed reactions from children, family members, and healthcare staff to gauge the effects MEDi^®^ was having and adjusted their practice accordingly. Rather than create ambitious goals, CLSs proceeded cautiously in learning the ‘who, what, where, when, why, and how’ of using MEDi^®^. Through this gradual process of gaining experience and insight, CLSs became increasingly skilled in leveraging potential uses of MEDi^®^.

To help organize, troubleshoot and encourage ongoing use of MEDi^®^, a CLS – the second author – was appointed as a leader. The initial assignments included understanding how the robots work, training the whole CLS team, and educating the hospital site about the best use of this technology. A quality improvement study (Farrier et al., 2019) conducted by a team of researchers at this hospital then guided the next phases of implementation locations, patient-care situations, and allocation of CLSs using the robot. Awareness of the robot throughout the hospital increased and referrals to the CLS team were received from areas outside of the units with which CLSs had historically been involved – partly due to interest in the robot and also due to program restructuring. Then other CLSs, especially those who worked in ambulatory clinics, became curious about the potential for MEDi^®^ in their interventions and began expressing interest in using MEDi^®^ in different ways. This group of CLSs makes up the majority of the participants in the current study because they are the ones who used MEDi^®^ most frequently during the first 5 years of implementation.

### Recruitment

In 2019, we purposely sampled this group of seven CLSs to ensure that participants were able to inform our understanding of implementing and sustaining use of MEDi^®^ as a hospital care innovation. The CLS team leader verbally introduced the study to the CLS team. The CLS lead for the MEDi^®^ robot project (second author) then sent them an email with further information and an invitation to contact her with questions or concerns. All seven CLSs contacted agreed to be interviewed. Participants were female and had been in practice as a CLS between 1.3 and 29 years, with an average of 17 years of practice experience, working in a variety of hospital clinics. The participant with the fewest years of experience as a CLS had completed her internship under the supervision of the leader of the project and regularly used MEDi^®^ in her interactions with patients. She also completed a fellowship with the broader CLS team and used MEDi^®^ frequently throughout this experience. Although her time as a CLS was short in comparison to some of the other participants in this study, she was very involved in the implementation of MEDi^®^, and we, therefore, deemed her to have the depth of experience to inform our goal of understanding how innovation can be implemented and sustained.

### Data Collection

Data were collected through semi-structured, individual interviews held in a private meeting room at the hospital where all the participants worked. Informed consent was discussed with and signed by participants. Participants were then asked a series of open-ended questions developed by Authors 1 and 2 to elicit the context and experience of using MEDi^®^. Participants were asked to begin by describing initial attempts to use MEDi^®^ and then their experiences up to and including their present use of MEDi^®^. Probes were used to elicit clarifications and depth of participant thoughts. The questions were focused on five topics. The first was about the adoption process, which consisted of questions such as, “Thinking back to your introduction of MEDi^®^, what were your impressions/feelings?” and “What was the adoption process like?” The second topic was about how using MEDi^®^ aligned with the role of the CLS and exemplar questions included, “How would you describe your role and how does MEDi^®^ fit with this role?” and “What types of goals can MEDi^®^ accomplish?” The third topic was about impact of using MEDi^®^ on the hospital environment and included, “How has MEDi^®^ affected the hospital environment?” and “Did you make any changes to your work/environment as a result of MEDi^®^?” The fourth topic was about CLSs’ insights into using MEDi^®^ with questions that included, “What are the most obvious advantages/disadvantages of MEDi^®^?” Finally, the fifth topic was about CLSs’ future outlook on the use of MEDi^®^, which was queried through questions such as, “What are the biggest challenges you foresee?”

Data collection interviews began with the first author interviewing the second author given that the second author is a CLS who has not only worked with, but also led initial implementation of, MEDi^®^. Authors 1 and 2 subsequently interviewed the other six participants together. These six interview participants know the second author as a colleague, and the first author as a researcher studying MEDi^®^. Interview duration ranged between 44 and 77 min, and averaged 55 min. All interviews were audiotaped and then transcribed verbatim by a research assistant.

Our narrative approach is inspired by Narrative Inquiry methodology which is rooted in generating findings that are the product of relationships between researchers and participants (Clandinin et al., 2009, 2018). As such, researchers and participants intentionally “co-compose” findings. Our efforts to co-compose findings were enhanced by collecting data from a participant who is both participant and co-author and also by employing members checking with all participants to invite and include their input on our early rounds of analysis.

### Ethical Considerations

Participants were aware of their relationship to the first and second authors. That is, the first author had been part of a research team that conducted the initial studies with MEDi^®^, presented the findings, and then introduced MEDi^®^ to the CLS team. The second author is a member of the CLS team and was the appointed lead in supporting CLSs in using MEDi^®^. These connections were fully disclosed to the Conjoint Health Research Ethics Board and to the senior administration at the hospital who approved this study. Through both the informed consent process and verbal instruction, participants were informed that their responses would in no way affect these relationships. Participants were also told that they could choose not to answer any questions that made them uncomfortable.

### Data Analysis

We conducted a conventional content analysis through which we scaled down our data and identified essential consistencies and meanings evidenced in participant accounts (Creswell and Poth, 2018). This entailed Authors 1 and 2 immersing themselves in the data, reducing data to preliminary codes according to interview topic area, and creating a corresponding codebook. Then, two research assistants coded the complete data set and modified the codebook, with input and additional coding completed by Authors 1 and 2. Author 3, an experienced qualitative researcher, triangulated analysis through iterative rounds of email discussions with Authors 1 and 2. Through these rounds, we achieved consensus about how best to condense, elaborate and refine codes that call attention to how participant implementation and sustaining of MEDi^®^ into their daily practice constitutes hospital pediatric care innovation.

## Results

We generated three main themes of findings (Table 1): (1) Adopting MEDi^®^ as part of Daily Practice; (2) Using MEDi^®^ in Alignment with the CLS role; and (3) MEDi^®^’s Impact on the Hospital Environment. Our first theme of Adopting MEDi^®^ as part of Daily Practice is a profiling of the ups and downs of CLSs’ experiences of learning about and implementing MEDi^®^. Our second theme of Using MEDi^®^ in Alignment with the CLS role is about how CLSs leveraged MEDi^®^ in their practice, and we found three sub-themes of evidence of CLS efforts that include: (a) Using MEDi^®^ to expand CLSs’ repertoire and effectiveness; (b) Using MEDi^®^ to support children to be leaders in their hospital care; and (c) Working around the extra steps and limits involved in using MEDi^®^. Our third theme of MEDi^®^’s Impact on the Hospital Environment is comprised of two subthemes, the first of which is (a) Bringing smiles and comfort to patients, which is about MEDi^®^’s impact on individual patient experiences; our second subtheme is (b) Contributing to a view of the hospital as a fun and friendly environment and is about MEDi^®^’s broader impact on perceptions of the hospital.

**TABLE 1:** Summary of main and sub-themes.

Themes	Description
(1) Adoption	Process of implementing MEDi^®^ into daily practice
(2) Alignment with CLS role	How used MEDi^®^ in alignment with CLS role
(a) CLS role expansion	How used MEDi^®^ to expand CLS role
(b) CLS primary role support	How used MEDi^®^ for primary role to support children
(c) Working alongside limits	How used MEDi^®^ given its limitations
(3) Hospital impact	Impact of MEDi^®^ on hospital environment
(a) Impact on individual patients	Impact of MEDi^®^ on patients in form of comfort
(b) Impact on hospital perceptions	Impact of MEDi^®^ on creating affectionate environment

### Adopting MEDi^®^ as Part of Daily Practice

Child life specialists expressed a variety of thoughts and emotions about the process of adopting MEDi^®^ as part of their daily practice. Positive and negative emotions were experienced upon the introduction of MEDi^®^ with several CLSs communicating their regard for MEDi^®^ as a cool and exciting addition to their practice. Some CLSs expressed having been confused and scared by the prospect of using MEDi^®^. Many reported initial thoughts of curiosity around how MEDi^®^ could be used and what might be possible by using MEDi^®^.

The process of adjusting to regular use of MEDi^®^ was often described by CLSs as trial and error with the greatest challenge being learning how to operate MEDi^®^. Indeed, one CLS indicated not always remembering how to operate MEDi^®^ and some CLSs expressed frustration and nervousness about remembering the steps of operating MEDi^®^. CLSs considered it important to learn what to do if MEDi^®^ did not work while they were with a patient to ensure that they could provide support to the patient while being careful not to damage MEDi^®^. CLS training provided by the second author in the form of instruction and verbal support were helpful in this learning. Although two CLSs had initially been reluctant to learn the technical aspects of how to use MEDi^®^, others indicated that this learning came naturally.

When asked what information they would have liked to have known when MEDi^®^ was first introduced, some CLSs indicated wishing they had known more about how MEDi^®^ worked, while others said they could have had more information about what MEDi^®^ can do. Several also noted that it was feasible to learn this information during the process of using MEDi^®^.

CLSs indicated that after gaining experience and familiarity with using MEDi^®^, they came to see MEDi^®^ as a beneficial and/or adaptable tool, and several reported feeling comfortable and enjoying using MEDi^®^. CLS1 summed up with the comment: “I’m just fascinated by what a fantastic tool it has been to connect with kids in a unique way. I always enjoy the smiles I see from kids when we when get MEDi^®^ dancing, singing.”

### Alignment With the CLS Role

The Child Life Specialist’s role includes supporting children to help them manage pain and prepare them for medical procedures. CLSs also advocate for children and normalize, as well as validate, children’s experiences. In describing how MEDi^®^ fits with their roles and responsibilities, CLSs supply evidence, which we organized according to how MEDi^®^ was used: (1) Using MEDi^®^ to expand CLS repertoire and effectiveness; (2) Using MEDi^®^ to support children to be leaders in their hospital care; and (3) Working around the extra steps and limits involved in using MEDi^®^.

#### Using MEDi^®^ to Expand CLSs’ Repertoire and Effectiveness

As well as describing working with MEDi^®^ as being inherently “fun” or “cool”, CLSs provided examples of how MEDi^®^ expanded their repertoire of tools and effectiveness in accomplishing the goals of their CLS role. CLSs appreciated having MEDi^®^ as an option to use in providing therapeutic support, and two CLSs said they use MEDi^®^ as a first option. MEDi^®^’s appeal as an option included the ways MEDi^®^ could help CLSs build connections with children and their parents. CLS1 called MEDi^®^ “a good conversation starter.” Parents were often intrigued by their children’s reactions to MEDi^®^ and thought MEDi^®^ provided a positive distraction to help children cope with painful medical procedures. For example, CLS2 stated:

And parent wise, we had some of the parents of the younger kids – they can see their child getting engaged with MEDi^®^ and being distracted in the benefits of it. Of course, it’s going to be positive.

MEDi^®^ also helped CLSs connect with parents through parent interest in the technological side of MEDi^®^. CLS2 noted:

A lot of the dads, so to speak, were intrigued by the scientific side of it all as well. I never really would say any of the parents were not positive about it. Yeah, they all thought it was neat.

Child life specialists pointed out that, not unlike parents, children take a scientific interest in MEDi^®^. Yet children often go beyond a scientific interest to form a human-like connection with MEDi^®^ replete with features of friendship and companionship that help children feel validated and supported. Thus, the CLS repertoire was expanded as CLSs could provide support and validation to children by personally connecting with them and also by facilitating their connecttion with MEDi^®^. CLS5 reflected:

I think it’d be a connection piece… Every time, like every single time, he [MEDi^®^] says someone’s name and says hello and uses their name, it’s like shock and awe and amazement and it’s fun to see that. That introduction in itself bridges to the next intervention that you want to do with him cuz, ‘Wait. He said my name.’ You’re getting by just from that hello. So that’s really fun. Never get tired.

Correspondingly, CLSs spoke of MEDi^®^ as adding “a new way of interacting with children” (CLS1). CLS6 described her delight at watching children interact with MEDi^®^: “When you look at them and the way they’re responding to MEDi^®^ – it’s, it’s just pure joy, and it’s just fun and that’s exciting” (CLS6).

Child life specialists pointed out that MEDi^®^ can be adapted to a variety of situations including teaching children coping strategies, encouraging children to develop courage, and motivating and rewarding children for positive behaviors. CLS3 summed up how MEDi^®^ can contribute to CLSs’ effectiveness with her broad comment that MEDi^®^ “helps children cope throughout hospitalization. So, he helps make being in the hospital for kids easier. So, he can, he helps us do our job better and helps kids in the hospital.” CLS7 added dimension to how MEDi^®^ can help CLSs do their job well as she noted MEDi^®^’s potential to bring attention to CLSs/the work of CLSs: “I find it [MEDi^®^] a very exciting piece of our responsibilities here at the hospital. I think we get a lot of attention…It sort of increases our popularity for sure.”

Child life specialists were looking forward to more behaviors being programmed into MEDi^®^ with more advances in technology in response to the increasingly complex and unique needs of pediatric patients. Several were planning to use MEDi^®^ more in the future and one was looking forward to conducting more research on the phenomenon of robotics in pediatric care.

#### Using MEDi^®^ to Support Children to Be Leaders in Their Hospital Care

As part of helping children feel connected and better able to manage their hospital experiences, CLSs found that MEDi^®^ could support children to be leaders in their hospital care. CLS1 described children as finding their own ways of using MEDi^®^ therapeutically:

I’m always amazed as to how the kids come up with different ways in which we can use the robot and how they sort of lead the way in finding a way it can be helpful and therapeutic to them.

MEDi^®^ as a tool for supporting children to be leaders in their hospital care could have distinct meaning for children with autism or developmental delays. CLS7 described how a patient’s interaction with MEDi^®^ allowed CLS7 to remain in the background:

I personally feel the greatest impact in general that MEDi^®^ has had has been with children on the autism spectrum and children with developmental delays. This boy now, it’s just part of his routine that he shows up at the hospital, he meets MEDi^®^, pushes the cart, you know, to the clinic that he needs to go to and he’s almost taken ownership. We’re sort of, I feel like I take the back burner with this young man.

CLS1 shared a favorite story of MEDi^®^ that involved helping a child with autism lead his dental care experience:

Okay so there’s a little boy…who has to come regularly to the dental clinic. The goal is to have a checkup, a regular check-up of his teeth without sedation. He has autism and the dental clinic does something called happy visits where they have the child come in and just practice getting familiar with the equipment in the environment and the different people and he would not even walk into the clinic the first time… It would take a lot of coaxing to even get him into the room. He would not sit on the chair…So someone suggested that maybe we try integrating MEDi^®^ into the happy visit. So, we put MEDi^®^ in the dental clinic room. This was my very first meeting of [the boy] in the waiting room and showed him a picture of MEDi^®^ and then asked him if he would like to go and meet the robot in the room and he just walked into the room and wanted to have MEDi^®^ set up so that he could touch him if he wanted to, he could lift him, he could put MEDi^®^ in the chair… and then he sat on the chair and held MEDi^®^ on the chair. The dental assistant raised the chair up and down to get familiar with that and we basically went through all the steps involved in what would be a real checkup and he cooperated with everything. His mom took video throughout the whole thing. She just could not believe that that was her son doing all this.

While CLSs remain key supports and advocates for children, MEDi^®^ was a means through which children could experience a greater sense of control and confidence in their experience.

#### Working Around the Extra Steps and Limits Involved in Using MEDi^®^

To expand their repertoire and effectiveness, CLSs needed to work around extra steps required to use MEDi^®^. These tasks included practical issues of storing and retrieving MEDi^®^ robots as well as remembering how to operate them. CLSs stated that they wished MEDi^®^ was more autonomous and responsive in conversation. One CLS indicated that setup to use MEDi^®^ takes longer than when using other tools, while other CLSs qualified that by using MEDi^®^, procedures with children progress more quickly thereby reducing the overall amount of time needed. At the same time, occurrences of technical glitches with MEDi^®^ could take extra time, disrupt the flow of procedures and demand that CLSs think quickly to work around glitches that could not be resolved in the moment. CLS3 described the challenge this way:

I think the only disadvantage using MEDi^®^ would be when he has any technical difficulties during a procedure. So, when he has technical difficulties, when it’s not a procedure, it’s okay cuz you kind of laugh and you’re like, ‘Oh MEDi^®^’s in the hospital too, like he’s a little, a little hurt right now.’ But just maybe during the procedure, if he’s supposed to be a distraction and something happens and you’re kind of frantically being like, ‘Oh no, just wait.’ I think that would be the only disadvantage.

Related accommodations by CLSs occurred during “downtimes” when the MEDi^®^ robots were shipped away for maintenance and repair.

Child life specialists also took extra steps to plan their use of MEDi^®^ that involved understanding and discerning situations where using MEDi^®^ would not be helpful. CLSs described negative reactions to MEDi^®^ – such as fear – shown by some younger children. Other children did not seem to engage with MEDi^®^ or might even develop a negative association between MEDi^®^ arriving and a painful medical procedure occurring. In other instances, MEDi^®^ might contribute unwelcome distraction and noise. For example, CLS5 stated:

I think mom had met MEDi^®^ and wanted us to use MEDi^®^ but then MEDi^®^ kind of did end up being that extra noise and extra confusion in the room. And after it wasn’t, which sometimes happens where if he’s not distracting and he’s dancing then it can be extra noise at times in the room. And so, I think for her it just added. We needed more calm which is not all on MEDi^®^ cuz I’m the one running MEDi^®^ and stuff.

In the example above, CLS3 had to accommodate on two levels: (1) that MEDi^®^ was a confusing distraction during a particular procedure, and (2) that CLS3 had been ill advised by a hopeful parent to include MEDi^®^ in this particular procedure. Indeed, a key accommodation facing CLSs is managing expectations of parents who might overestimate MEDi^®^’s potential to quickly make children more cooperative. CLSs also managed overestimations by other healthcare providers about MEDi^®^’s potential to make children more cooperative. CLS1 shared an example where she noted that although MEDi^®^ could help children who were particularly nervous, other healthcare providers expected MEDi^®^ to help a nervous child become immediately or perfectly cooperative. In CLS1’s example, although a child was a great deal more cooperative, an anesthesiologist concluded that MEDi^®^ had not worked: “It was a billion times better than any prior procedure, but the anesthesiologist concluded that the robot didn’t work.”

When MEDi^®^ did not have the desired effect, or was perceived as not having the desired effect, CLSs could be left feeling judged by parents or other healthcare providers. CLS3 brought together ideas of MEDi^®^’s potential along with MEDi^®^’s limits:

I think for some kids MEDi^®^ is so influential because other solutions aren’t robots and aren’t something alive like not alive… for some children I think MEDi^®^ would solve a lot of the problems they are having. And for some kids it may not be MEDi^®^, it may be something else. I think he’s one of the solutions to some of the problems kids have.

### MEDi^®^’s Impact on the Hospital Environment

When asked about the impact MEDi^®^ has on the hospital environment, CLSs identified groups who particularly enjoy MEDi^®^: parents, children with autism spectrum disorder, and children who were not too young (i.e., not less than about 4 years-of-age) and children who are not too old (not older than early teenagers). CLSs went on to emphasize MEDi^®^’s impact on the hospital in terms of (1) bringing smiles and comfort to patients; and (2) contributing to a view of the hospital as a fun and friendly environment.

#### Bringing Smiles and Comfort to Patients

CLSs described the power of MEDi^®^ to make children smile. CLS7 stated:

There is a young boy right now that I’m seeing in dental clinic and he talks to MEDi^®^ like MEDi^®^ is an actual human being and wants to hold MEDi^®^ and yeah. He just makes kids smile.

Child life specialists spoke in terms of how MEDi^®^ could make children suddenly happy and cooperative, how children fell in love with MEDi^®^. The special relationship children formed with MEDi^®^ could make the hospital experience positive for the family.

As well as provoking smiles, MEDi^®^ was described as providing children with a sense of comfort and safety. CS7 spoke of the physical comfort children derive from MEDi^®^ as a “snuggle” (CLS7). CLSs showed how MEDi^®^ had become part of the hospital environment as they noted that children will request that MEDi^®^ visit them.

#### Contributing to a View of the Hospital as a Fun and Friendly Environment

Building on our earlier finding about how MEDi^®^ can contribute to expanding CLSs’ effectiveness by bringing attention to their work, they spoke of how MEDi^®^ helps create a view of the hospital as a fun and friendly environment. MEDi^®^ serves as a point of focus for people visiting the hospital. CLS7 noted that, “Families like seeing him in the hallway, you know, there’s lots of pointing and smiling.” CLS5 echoed this observation stating: “He turns heads for sure.” CLS3 expanded by describing MEDi^®^’s impact on perceptions of the hospital environment: “Kids aren’t just walking through the hospital, they walk through and see a robot, which makes it a fun and easy and friendly environment.”

MEDi^®^’s contributions to positive views of the hospital build on MEDi^®^’s interactive functions in supporting children through difficult procedures as CLS3 pointed out with her claim that MEDi^®^ is “such a great tool and resource that we can use to help kids and not be traumatized in the hospital and to change their outlook and view of what going to the hospital is.” CLS7 added a point about how MEDi^®^ has the effect of distinguishing their hospital from other hospitals: “I think he’s a very unique tool that we can use with patients that not every hospital offers.”

MEDi^®^’s contribution to views of the hospital as a fun and friendly environment is enhanced by how MEDi^®^ can effect relationships among staff members. For example, CLS2 stated:

When you walk down the hall…if you were pushing MEDi^®^ down the hallway, staff still got excited to see you like ‘Oh there’s that robot.’… I was in the elevator yesterday and the security guard hadn’t seen MEDi^®^ before and I explained a little bit and he just lit up and he’s like, ‘it makes me so happy to see this here for kids.’

Reflecting on MEDi^®^’s contribution to the hospital leaves CLSs pondering how MEDi^®^ might be implemented in other areas of healthcare: CLS6 stated “You know, I even think that we don’t have to just stick to pediatrics. I think it would work well in adult hospitals too.” CLSs suggested that volunteers could operate MEDi^®^ with patients for purposes of providing distraction.

## Discussion

Findings from this study demonstrate how frontline workers implemented a tangible long-term innovative approach to supporting children at the bedside. Through our themes we highlight how participants experienced and were able to overcome the challenges of learning how to use MEDi^®^ in their daily practice. We illustrate how participants were able to find ways of using MEDi^®^ to both comfort and encourage children to take a leadership role in their care and how their use of MEDi^®^ aligns with their roles and responsibilities as CLSs. We show participant views of how MEDi^®^ contributes to building a friendly hospital environment.

CLS shared various emotions about their initial experiences working with the robot which, became more predominantly positive over time. At the time of the interview, a sense of comfort, enjoyment, and even fascination were reported. Despite several years of observing MEDi^®^’s impact on children, CLSs continued to be amazed at how happy children felt with MEDi^®^. Negative emotions centered on remembering the steps to operate MEDi^®^. Thus, we recommend that implementation of new technology be accompanied with accessible and simple step-by-step instructions, even though these may not be needed for all users. An important finding with implications for implementing future innovations is that the CLSs seemed to gain more confidence in using MEDi^®^ when they had a plan for an alternate intervention if they could not operate MEDi^®^ at the time they needed it. Technical difficulties in operating a robot are not unique in this study (Song and Yamada, 2017). Thus, education about the innovation and ongoing support are also recommended.

CLS are responsible for comforting distressed children. They use various strategies to accomplish this goal such as educating children about medical procedures, distracting them during painful and frightening procedures, teaching them coping strategies, normalizing their experiences, and validating their feelings. CLSs explained that MEDi^®^ helps them in these goals by engaging children through an emotional connection. Indeed, a study in which therapists were asked about the types of functions they would like to see a robot be able to perform as part of their therapeutic support of children with autism, two recommendations identified programming a robot to identify both positive and negative patient emotions to promote the former and discourage the latter (Zubrycki and Granosik, 2016). Thus, professionals who work with children seem to be enthusiastic toward integrating a robot for therapeutic support. With the children’s attention on an emotional connection with MEDi^®^, the CLS can then play MEDi^®^ to act and talk in ways that comfort children. It appears that the CLSs shifted into a facilitative role where the interaction was more directly between the robot and child. In such interactions, the child was able to take the lead and create a sense of control over their experience at the hospital, which is normally dictated by the healthcare professionals. The CLSs’ thought process of how to accomplish these goals was not explored in the present study and is recommended for future research. These insights would help guide other CLSs in adopting this technology in their daily practice. What is clear, nevertheless, is that CLSs have the knowledge and skill to operate MEDi^®^ in a way that delights and comforts children. It was also noted that despite its many capabilities, MEDi^®^ must have an advocate who knows the child’s needs and addresses them by how he/she plays MEDi^®^. Simply put, the robot cannot replace the CLS.

In addition to emotional comfort, children reportedly experienced physical comfort and emotional safety with MEDi^®^. Additionally, children’s emotions such as sudden happiness and even feeling love for MEDi^®^ were described by CLSs. There are two primary explanations for this finding. First, studies in Human-Robot Interaction have found that people can attribute emotion to a robot (Novikova and Watts, 2015). Also, a robot’s eye gaze toward a person has been shown to facilitate feelings of intimacy (Admoni and Scassellati, 2017). Taken in combination, MEDi^®^’s features such as its ability to express emotion and share eye gaze with children may have evoked their strong pleasurable emotions – emotions that can subdue fear and anxiety that children may otherwise feel when in the hospital (Czarnecki et al., 2011). Observing these reactions in children and the excitement this creates in parents and hospital staff was described as “pure joy”, which seem to heighten CLSs’ enthusiasm for using MEDi^®^. In fact, some CLSs stated that MEDi^®^ is the first strategy they use when providing support to children. Given that excitement at work is associated with low levels of burnout (Sadovich, 2005), along with the risk for burnout experienced by CLSs (Holloway and Walling, 1990), involvement with implementing an innovative approach may have positive effects on CLSs’ job satisfaction. Thus, impact on the hospital environment was both felt, and created, by CLSs, children, their parents, and hospital staff via MEDi^®^.

This positive impact seemed to create high expectations about effectiveness. Having witnessed extreme positive reactions of children toward MEDi^®^, healthcare staff began to maintain this expectation for all children. Also, when staff were unaware of children’s previous extreme reactions of distress, children’s reduction in distress with MEDi^®^ was interpreted as ineffective if children were less than fully cooperative. We recommend that CLSs manage these expectations by explaining to staff that each child reacts differently to MEDi^®^ and there is no guaranteed positive or perfect impact for any particular approach.

### Limitations and Future Research

Despite the unique vantage point of our participants, there are several potential biases in our study. Participants were all female and recruited at only one hospital. We recommend that other hospitals employing the use of this or other robots for similar purposes be included in future research to understand other experiences of implementing this type of innovation. Also, the sample is small and includes a co-author participant. However, an advantage of the present study is that all of the CLSs with the most experience using MEDi^®^ contributed their perspectives about their considerable experience. Although they may have been motivated to share socially desirable responses in the interviews, they also shared some negative experiences and were explicitly encouraged to describe disadvantages. They may also have generated responses in the interview that could have been influenced by novelty effects of using a new innovation. However, the intervention had been implemented throughout the hospital for 5 years. Another potential bias is the reliance on memory recall when asking participants to think back to their initial reactions to MEDi^®^. Future research can document the implementation process as it progresses. Also, a bias exists in terms of the familiar relationship between the researchers and the participants. Despite our attempts to provide full disclosure, maintain awareness of our biases, and hold them aside, future studies are needed with a researcher from outside the hospital community to explore similar or different findings.

## Conclusion

Findings from this study reveal the challenges and triumphs of implementing an innovative and technologically advanced approach to bedside pediatric care in a hospital environment. It is not surprising that CLSs experienced a challenge in learning how to operate a robot, as it is such an innovative device to be found in a hospital. However, with some training and support, they seemed to develop mastery to the extent that it became the first strategy they currently use. The implementation of this innovation clearly became a tangible reality, as opposed to a lofty aspiration, when all of the CLSs in our study recommended MEDi^®^ for other hospitals. Moreover, the effect of feeling “popular” suggests that there could be many unanticipated benefits that outweigh the risk of trying a new innovation.

## Data Availability Statement

The only data that can be made available is the codebook. The health authority will not release transcripts as they contain patient data. Requests to access the datasets should be directed to the corresponding author.

## Ethics Statement

The studies involving human participants were reviewed and approved by University of Calgary Conjoint Health Research Ethics Board. The participants provided their written informed consent to participate in this study.

## Author Contributions

TB and JP contributed to all aspects of the research. BL contributed to the analysis and write-up. All authors contributed to the article and approved the submitted version.

## Conflict of Interest

TB is commercializing the MEDi^®^ robot and developed the behaviors in the MEDi^®^ software. This relationship is disclosed at all research presentations and documented on the consent form. The remaining authors declare that the research was conducted in the absence of any commercial or financial relationships that could be construed as a potential conflict of interest.

## Footnotes

1 http://doc.aldebaran.com/2-1/family/robots/index_robots.html

## References

[B1] Admoni H. Scassellati B. (2017). Social eye gaze in human-robot interaction: a review. *J. Hum. Robot. Interact.* 6 25–63. 10.5898/JHRI.6.1.Admoni 28396741

[B2] Association of Child Life Professionals (2021). Available at: https://www.childlife.org/the-child-life-profession

[B3] Baxter P. Jack S. (2008). Qualitative case study methodology: study design and implementation for novice researchers. *Qual. Rep.* 13 544–559. 10.46743/2160-3715/2008.1573

[B4] Beran T. N. Ramirez-Serrano A. Kuzyk R. Fior M. Nugent S. (2011a). Understanding how children understand robots: animism in the 21st century. *Int. J. Human Comput. Stud.* 69 539–550.

[B5] Beran T. N. Ramirez-Serrano A. Kuzyk R. Nugent S. Fior M. (2011b). Would children help a robot in need? *Int. J. Soc. Robot.* 3 83–93.

[B6] Beran T. N. Ramirez-Serrano A. Vanderkooi O. Kuhn S. (2013). Reducing children’s distress towards flu vaccinations: a novel and effective use of humanoid robotics. *Vaccine* 31 2772–2777. 23623861 10.1016/j.vaccine.2013.03.056

[B7] Beran T. N. Ramirez-Serrano A. Vanderkooi O. Kuhn S. (2015). Humanoid robotics in health care: an exploration of children’s and parents’ emotional reactions. *J. Health Psychol.* 20 984–989. 24140615 10.1177/1359105313504794

[B8] Clandinin D. Caine V. Lessard S. (2018). *The Relational Ethics of Narrative Inquiry.* New York, NY: Routledge.

[B9] Clandinin D. Murphy M. Huber J. Orr A. (2009). Negotiating narrative inquiries: living in a tension-filled midst. *J. Educ. Res.* 103 81–90. 10.1080/00220670903323404

[B10] Cresswell K. Cunningham-Burley S. Sheikh A. (2018). Health care robotics: qualitative exploration of key challenges and future directions. *J. Med. Internet Res.* 20:e10410. 10.2196/10410 PMC6053611 29973336

[B11] Creswell J. Poth C. (2018). *Qualitative Inquiry and Research Design*, 4th Edn. London: Sage.

[B12] Czarnecki M. Turner H. Collins P. Doellman D. Wrong S. Reynolds J. (2011). Procedural pain management: a position statement with clinical practice recommendations. *Pain Manage. Nurs.* 12 95–111. 10.1016/j.pmn.2011.02.003 21620311

[B13] Di Nuovo A. Bamforth J. Daniela C. Sage K. Ibbotson R. Clegg J. (2020). “An explorative study on robotics for supporting children with autism spectrum disorder during clinical procedures,” in *Proceedings of the ACM/IEEE International Conference on Human-Robot Interaction*, Christchurch. 10.1145/3371382.3378277

[B14] do Carmo Caccia-Bava M. Guimaraes V. Guimaraes T. (2009). Testing some major determinants for hospital innovation success. *Int. J. Health Care Qual. Assur.* 22 454–470. 19725367 10.1108/09526860910975571

[B15] Farrier C. Pearson J. Beran T. N. (2019). Reducing children’s fear and pain towards medical procedures: a nonrandomized trial with a humanoid robot. *Can. J. Nurs. Res.* 10.1177/0844562119862742 [Epub ahead of print]. 31318580

[B16] Fior M. Nugent S. Beran T. N. Ramirez-Serrano A. Kuzyk R. (2010). Children’s relationships with robots: robot is child’s new friend. *J. Phys. Agents* 4 9–17.

[B17] Holloway D. Walling C. (1990). Burnout in child life specialists: the relation of role stress. *Child. Health Care* 19 10–18.

[B18] Jibb L. A. Birnie K. A. Nathan P. C. Beran T. N. Hum V. Charles Victor J. (2018). Using the MEDiPORT humanoid robot to reduce procedural pain and distress in children with cancer: a pilot randomized controlled trial. *Pediatr. Blood Cancer* 65:e27242. 10.1002/pbc.27242 29893482

[B19] Lysaker P. H. Buck K. D. (2006). Narrative enrichment in the psychotherapy for persons with schizophrenia: a single case study. *Issues Ment. Health Nurs.* 27 233–247. 10.1080/01612840500502676 16484168

[B20] MacNeil M. Koch M. Kuspinar A. Juzwishin D. Lehoix P. Stolee P. (2019). Enabling health technology innovation in Canada: barriers and facilitators in policy and regulatory processes. *Health Policy* 123 203–214. 30352755 10.1016/j.healthpol.2018.09.018

[B21] Maltese M. Henrich D. (2019). *The Challenge and Promise of Pediatric Device Innovation. Med Device Online.* Available online at: https://www.meddeviceonline.com/doc/the-challenge-and-promise-of-pediatric-device-innovation-0001 (accessed January 22, 2021).

[B22] Manaloor R. Ali S. Ma K. Sivakumar M. Vandermeer B. Beran T. (2019). Humanoid robot-based distraction to reduce pain and distress during venipuncture in the pediatric emergency department: a randomized controlled trial. *Paediatr. Child Health* 24(Suppl. 2), e43. 10.1093/pch/pxz066.112

[B23] Manesh S. A. Beran T. N. Sharlin S. Greenberg S. (April, 2014). “Medi, human robot interaction in pediatric health. Video Showcase at the ACM CHI Conference, Toronto,” in *Proceedings of the CHI ‘14 Extended Abstracts on Human Factors in Computing Systems (CHI EA ’14)*, (New York, NY: Association for Computing Machinery), 153–154. 10.1145/2559206.2579529

[B24] Mettler T. Sprenger M. Winter R. (2017). Service robots in hospitals: new perspectives on niche evolution and technology affordances. *Eur. J. Inf. Syst.* 26 451–468.

[B25] Mubin O. Khan A. Obaid M. (2016). “#naorobot: exploring Nao discourse on Twitter,” in *Proceedings Of The 28Th Australian Computer-Human Interaction Conference*, Launceston, 155–159. 10.1145/3010915.3011002

[B26] Nembhard I. Alexander J. Hoff T. Ramanujam R. (2019). Why does the quality of health care continue to lag? Insights from management research. *Acad. Manage. Perspect.* 23 24–42.

[B27] Novikova J. Watts L. (2015). Towards artificial emotions to assist social coordination in HRI. *Int. J. Soc. Robot.* 7 77–88. 10.1007/s12369-014-0254-y

[B28] Pearson J. R. Beran T. N. (2018). “The future is now: using humanoid robots in child life practice,” in *Handbook of Medical Play Therapy and Child Life*, ed. Rubin L. (New York, NY: Routledge), 351–372.

[B29] Rabbitt S. M. Kazdin A. E. Scassellati B. (2015). Integrating socially assistive robotics into mental healthcare interventions: applications and recommendations for expanded use. *Clin. Psychol. Rev.* 35 35–46. 10.1016/j.cpr.2014.07.001 25462112

[B30] Rangachari P. (2018). Innovation Implementation in the context of hospital QI: lessons learned and strategies for success. *Innov. Entrep. Health* 5 1–14. 29546884 10.2147/IEH.S151040 PMC5849396

[B31] Sadovich J. (2005). Work excitement in nursing: an examination of the relationship between work excitement and burnout. *Nurs. Econ.* 23 91–96. 15881495

[B32] Song S. Yamada S. (2017). “Expressing emotions through color, sound, and vibration with an appearance-constrained social robot,” in *Proceedings of the 12th ACM/IEEE International Conference on Human-Robot Interaction*, Vienna, 2–11.

[B33] Zuber C. Weberg D. (2020). Frameworks for leading frontline innovation in health care: failure, microclimates, and leadership. *Nurse Lead.* 18 290–295. 10.1016/j.mnl.2020.03.005

[B34] Zubrycki I. Granosik G. (2016). Understanding therapists’ needs and attitudes towards robotic support: the Roboterapia Project. *Int. J. Soc. Robot.* 8 553–563. 10.1007/s12369-016-0372-9

